# Auto-Abs neutralizing type I IFNs in patients with severe Powassan, Usutu, or Ross River virus disease

**DOI:** 10.1084/jem.20240942

**Published:** 2024-11-01

**Authors:** Adrian Gervais, Paul Bastard, Lucy Bizien, Céline Delifer, Pierre Tiberghien, Chaturaka Rodrigo, Francesca Trespidi, Micol Angelini, Giada Rossini, Tiziana Lazzarotto, Francesca Conti, Irene Cassaniti, Fausto Baldanti, Francesca Rovida, Alessandro Ferrari, Davide Mileto, Alessandro Mancon, Laurent Abel, Anne Puel, Aurélie Cobat, Charles M. Rice, Dániel Cadar, Jonas Schmidt-Chanasit, Johannes F. Scheid, Jacob E. Lemieux, Eric S. Rosenberg, Marianna Agudelo, Stuart G. Tangye, Alessandro Borghesi, Guillaume André Durand, Emilie Duburcq-Gury, Braulio M. Valencia, Andrew R. Lloyd, Anna Nagy, Margaret M. MacDonald, Yannick Simonin, Shen-Ying Zhang, Jean-Laurent Casanova

**Affiliations:** 1 Laboratory of Human Genetics of Infectious Diseases, Necker Branch, Institut National de la Santé et de la Recherche Médicale (INSERM) U1163, Necker Hospital for Sick Children, Paris, France; 2 Imagine Institute, Paris Cité University, Paris, France; 3 St. Giles Laboratory of Human Genetics of Infectious Diseases, Rockefeller Branch, Rockefeller University, New York, NY, USA; 4 Pediatric Hematology-Immunology and Rheumatology Unit, Necker Hospital for Sick Children, Assistance Publique-Hôpitaux de Paris (AP-HP), Paris, France; 5 Établissement Français du Sang, La Plaine Saint-Denis, France; 6 Faculty of Medicine, School of Biomedical Sciences, UNSW Australia, Sydney, Australia; 7 Neonatal Intensive Care Unit, San Matteo Research Hospital, Pavia, Italy; 8 Microbiology Unit, IRCCS Azienda Ospedaliero-Universitaria di Bologna, Bologna, Italy; 9 Department of Medical and Surgical Sciences, Section of Microbiology, University of Bologna, Bologna, Italy; 10 Pediatric Unit, IRCCS Azienda Ospedaliero-Universitaria di Bologna, Bologna, Italy; 11 Department of Medical and Surgical Sciences, Alma Mater Studiorum, University of Bologna, Bologna, Italy; 12 Department of Clinical, Surgical, Diagnostic and Pediatric Sciences, University of Pavia, Pavia, Italy; 13 Microbiology and Virology Department, Fondazione IRCCS Policlinico San Matteo, Pavia, Italy; 14 Laboratory of Clinical Microbiology, Virology and Bioemergencies, Luigi Sacco Hospital, ASST Fatebenefratelli Sacco, Milan, Italy; 15 Laboratory of Virology and Infectious Disease, The Rockefeller University, New York, NY, USA; 16 Bernhard Nocht Institute for Tropical Medicine, Hamburg, Germany; 17 Faculty of Mathematics, Informatics and Natural Sciences, University of Hamburg, Hamburg, Germany; 18 Massachusetts General Hospital and Harvard Medical School, Boston, MA, USA; 19 Laboratory of Molecular Immunology, The Rockefeller University, New York, NY, USA; 20 Garvan Institute of Medical Research, Darlinghurst, Australia; 21 Faculty of Medicine and Health, School of Clinical Medicine, University of New South Wales Sydney, Sydney, Australia; 22 School of Life Sciences, Swiss Federal Institute of Technology, Lausanne, Switzerland; 23 National Reference Center for Arboviruses, Inserm-IRBA, Marseille, France; 24 Unité des Virus Émergents (UVE: Aix-Marseille Univ-Corsica Univ-IRD 190-Inserm 1207-IRBA), Marseille, France; 25 Intensive Care Unit, Saint Philibert Hospital, Lille Catholic Hospitals, Lille, France; 26 The Kirby Institute, UNSW Australia, Sydney, Australia; 27 National Reference Laboratory for Viral Zoonoses, National Public Health Center, Budapest, Hungary; 28 Pathogenesis and Control of Chronic and Emerging Infections, University of Montpellier, INSERM, EFS, Montpellier, France; 29 Howard Hughes Medical Institute, New York, NY, USA; 30 Department of Pediatrics, Necker Hospital for Sick Children, AP-HP, Paris, France

## Abstract

Arboviral diseases are a growing global health concern. Pre-existing autoantibodies (auto-Abs) neutralizing type I interferons (IFNs) can underlie encephalitis due to West Nile virus (WNV) (∼40% of patients) and tick-borne encephalitis (TBE, due to TBE virus [TBEV]) (∼10%). We report here that these auto-Abs can also underlie severe forms of rarer arboviral infections. Auto-Abs neutralizing high concentrations of IFN-α2, IFN-β, and/or IFN-ω are present in the single case of severe Powassan virus (POWV) encephalitis studied, two of three cases of severe Usutu virus (USUV) infection studied, and the most severe of 24 cases of Ross River virus (RRV) disease studied. These auto-Abs are not found in any of the 137 individuals with silent or mild infections with these three viruses. Thus, auto-Abs neutralizing type I IFNs underlie an increasing list of severe arboviral diseases due to Flaviviridae (WNV, TBEV, POWV, USUV) or Togaviridae (RRV) viruses transmitted to humans by mosquitos (WNV, USUV, RRV) or ticks (TBEV, POWV).

## Teaser

Infections with arboviruses Powassan virus (POWV), Usutu virus (USUV), or Ross River virus (RRV) are rare but can cause severe disease. We report autoantibodies neutralizing type I IFNs in patients with POWV encephalitis, severe USUV infection, and severe RRV infection.

## Introduction

Arboviral diseases are transmitted to humans by mosquitos or, more rarely, by ticks (Davis et al., 2008). There are at least 150 human-tropic arboviruses belonging to the Togaviridae and Flaviviridae families of RNA viruses (Madewell, 2020). The range of clinical presentations of arboviral infections is vast. Most individuals have silent or benign infections, whereas a few suffer from life-threatening diseases (Pierson and Diamond, 2020). Over the last few decades, well-known emerging and re-emerging arboviral diseases have become a growing threat health worldwide (Gould and Solomon, 2008; Wilder-Smith et al., 2017). An estimated 700,000 deaths due to mosquito-borne viral infections alone occur yearly, constituting a major global public health burden (Ketkar et al., 2019). Virulence varies considerably between arboviruses, but interindividual clinical variability is also considerable for each of these viruses and remains unexplained, as in most common infectious diseases (Casanova, 2023; Casanova and Abel, 2024). We recently reported that pre-existing autoantibodies (auto-Abs) neutralizing type I interferons (IFNs) underlie ∼40% of West Nile virus (WNV) encephalitis cases (Gervais et al., 2023) and ∼10% of most severe forms of tick-borne encephalitis (TBE) (Gervais et al., 2024b). Auto-Abs neutralizing type I IFNs have been shown to underlie 5–20% of cases of life-threatening pneumonia due to severe acute respiratory syndrome coronavirus 2 (SARS-CoV-2) (Bastard et al., 2020, 2021a, 2021c, 2023, 2024), influenza (Zhang et al., 2022b), or Middle East respiratory syndrome (MERS) (Alotaibi et al., 2023) viruses, and about a third of severe adverse reactions to the attenuated live measles and yellow fever virus (YFV) vaccines (Bastard et al., 2021b). These findings have been replicated worldwide by many studies (Abers et al., 2021; Acosta-Ampudia et al., 2021; Akbari et al., 2023; Akbil et al., 2022; Arrestier et al., 2022; Bastard et al., 2021c; Busnadiego et al., 2022; Carapito et al., 2022; Chang et al., 2021; Chauvineau-Grenier et al., 2022; Credle et al., 2022; Eto et al., 2022; Frasca et al., 2022; Goncalves et al., 2021; Grimm et al., 2023; Hansen et al., 2023; Koning et al., 2021; Lamacchia et al., 2022; Lemarquis et al., 2021; Mathian et al., 2022; Meisel et al., 2021; Petrikov et al., 2022; Philippot et al., 2023; Pons et al., 2023; Raadsen et al., 2022; Savvateeva et al., 2021; Schidlowski et al., 2022; Saheb Sharif-Askari et al., 2023; Simula et al., 2022; Solanich et al., 2021; Soltani-Zangbar et al., 2022; Su et al., 2022; Troya et al., 2021; van der Wijst et al., 2021; Vanker et al., 2023; Vazquez et al., 2021; Wang et al., 2021; Ziegler et al., 2021). These auto-Abs are present in individuals of all ages in the general population, with a prevalence increasing from 0.3% to 1% in individuals under 65 years of age to 4–7% in individuals over 65 years of age (Bastard et al., 2021a).

In this context, we focused on three arboviral infections that are relatively rare in humans: Powassan virus (POWV), Usutu virus (USUV), and Ross River virus (RRV) infections. Most humans infected with these viruses do not develop symptoms or signs of sickness (Ashraf et al., 2015; Dobler, 2010; Harley et al., 2001; Hermance and Thangamani, 2017; Clé et al., 2019; Russell, 2002). POWV is a neurotropic orthoflavivirus transmitted by ticks in North America (Bassam et al., 1999; Hermance and Thangamani, 2017). The seroprevalence of POWV varies significantly between studies, ranging from 0.5% to 3% (Frost et al., 2017; Vahey et al., 2022). An estimated 23% of infections in New Jersey were considered severe, but with a bias toward older people and people reporting tick bites (Vahey et al., 2022). In another study on younger patients with no known history of tick bites, no severe cases were found among the dozen individuals infected (Frost et al., 2017). Fewer than 50 symptomatic cases are reported each year in the United States and almost all these cases are severe, with 60% occurring in people over the age of 60 years (CDC, 2024). Another neurotropic orthoflavivirus, USUV, is transmitted by mosquitoes in Africa and Europe (Ashraf et al., 2015; Clé et al., 2019; Nikolay et al., 2011). In Europe, the estimated seroprevalence for USUV varies considerably, ranging from 0.02% to 3% (Cadar and Simonin, 2022). Just over 100 symptomatic cases were reported in Europe between 2016 and 2021 (European Centre for Disease Prevention and Control, 2023), including about 30 severe neurological forms (meningitis, encephalitis, or meningoencephalitis) (Cadar and Simonin, 2022). The final virus considered here, RRV, is an arthritogenic alphavirus endemic to Oceania, where ∼4,000 symptomatic cases—typically presenting with fever and polyarthralgia or polyarthritis—are reported each year (Yuen and Bielefeldt-Ohmann, 2021). RRV has a median seroprevalence of 19% in endemic regions (Madzokere et al., 2022). No fatal cases of RRV infection have ever been reported and RRV-infected patients usually recover spontaneously or following primary care interventions (Harley et al., 2001; Russell, 2002). We hypothesized that auto-Abs neutralizing type I IFNs (Bastard et al., 2024; Casanova et al., 2024; Hale, 2023) might underlie at least some cases of severe disease due to these three arboviruses.

## Results and discussion

### Auto-Abs neutralizing IFN-ω in a patient with severe POWV encephalitis

**Figure 1.**
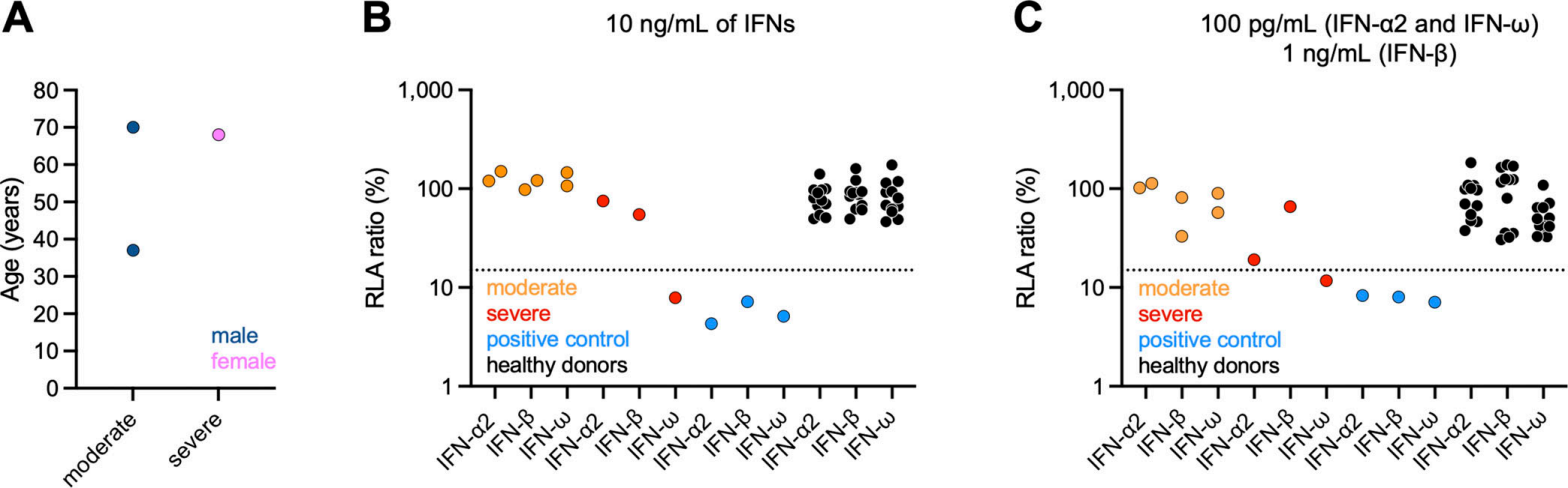
**Auto-Abs neutralizing type I IFNs in individuals infected with POWV. (A)** Age and sex distribution of the patients according to POWV disease severity. **(B and C)** Luciferase-based neutralization assay to detect auto-Abs neutralizing 10 ng/ml IFN-α2, IFN-ω, or IFN-β (B), or 100 pg/ml IFN-α2 and IFN-ω or 1 ng/ml IFN-β (C). The positive control (blue) was plasma from a patient with RAG1 deficiency known to carry auto-Abs neutralizing IFN-α2, IFN-ω, and IFN-β at a concentration of 10 ng/ml. Plasma samples from healthy donors (black) were obtained from individuals from the general population without auto-Abs neutralizing type I IFNs. HEK293T cells were transfected with (1) a plasmid containing the firefly luciferase gene under the control of an ISRE-containing promotor and (2) a plasmid containing the *Renilla* luciferase gene. The cells were then treated with type I IFNs in the presence of 10% plasma or serum from patients or controls, and RLA was calculated by dividing firefly luciferase activity by *Renilla* luciferase activity. An RLA <15% of the median RLA for healthy controls was considered to correspond to neutralizing activity (dotted line; Bastard et al., 2021a). The samples of the POWV patients were tested twice and the associated datapoints represent the mean RLA of these independent duplicates.

We investigated three patients with POWV disease: two men, aged 37 (P1) and 70 (P2) years, who were hospitalized for a moderate form of the disease with almost complete recovery, and a 68-year-old woman (P3) hospitalized for severe encephalopathy resulting in acute respiratory failure (Fig. 1 A). This patient developed chronic respiratory failure with ventilation dependence and experienced multiple complications, resulting in her death ∼1 year after POWV disease. Plasma samples were obtained from these patients during the first few days after symptom onset (P3) or after the illness (P1 and P2). The diagnosis of viral infection was based on positive results for the detection of anti-POWV IgM in the blood (and CSF for P3) and for a plaque reduction neutralization test (PRNT) against POWV. All three cases originated from and lived in the United States and their ancestry was unknown, as was their medical history, with the exception of POWV infection. Using a previously described luciferase-based neutralization assay (Bastard et al., 2021a), we tested 1:10 dilutions of serum or plasma from all subjects for the neutralization of high (10 ng/ml) or low (100 pg/ml) concentrations of non-glycosylated IFN-α2 and/or IFN-ω, and high (10 ng/ml) or intermediate (1 ng/ml) concentrations of glycosylated IFN-β (Fig. 1, B and C). No neutralization of any of the IFNs tested was observed with plasma samples from P1, P2, or any of the healthy donors. By contrast, plasma from P3-neutralized high and low concentrations of IFN-ω, like plasma from a RAG1-deficient patient known to have neutralizing auto-Abs against IFN-α2, IFN-β, and IFN-ω used as a positive control (Fig. 1, B and C). Unfortunately, the small sample volumes available precluded the testing of auto-Ab levels by another method (e.g., ELISA). Overall, neither of the two cases of moderate POWV disease tested (P1 and P2) displayed any detectable neutralization of type I IFNs, whereas such neutralization was observed for the only case of severe POWV disease tested (P3).

### Auto-Abs neutralizing IFN-α2, -β, and -ω in two patients with severe USUV disease

**Figure 2.**
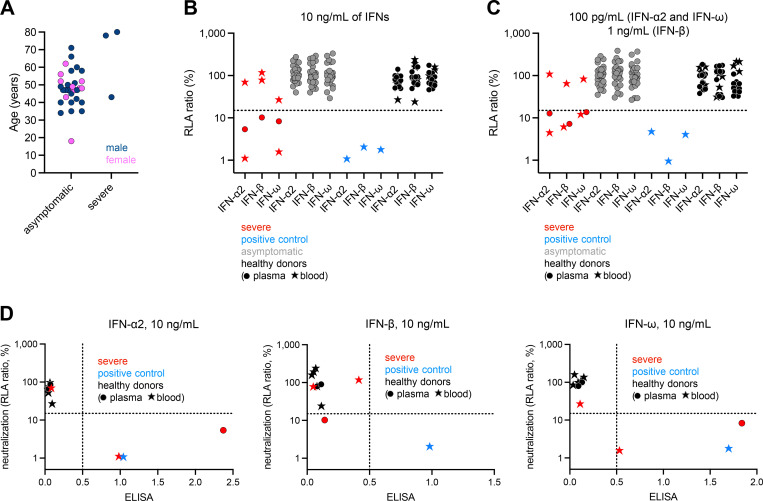
**Auto-Abs neutralizing type I IFNs in individuals infected with USUV. (A)** Age and sex distribution of the patients according to USUV disease severity. **(B and C)** Luciferase-based neutralization assay to detect auto-Abs neutralizing 10 ng/ml IFN-α2, IFN-ω, or IFN-β (B), or 100 pg/ml IFN-α2 and IFN-ω or 1 ng/ml IFN-β (C). The positive control (blue) was plasma from a patient with RAG1 deficiency known to carry auto-Abs neutralizing IFN-α2, IFN-ω, and IFN-β at a concentration of 10 ng/ml. Healthy donor plasma (black dots) and whole blood (black stars) samples were tested as negative controls; whole blood was tested because we had only whole-blood samples available for two of the three severe USUV cases. The asymptomatic cases (gray) tested positive for anti-USUV Abs during a blood donation but did not report symptomatic disease. HEK293T cells were transfected with (1) a plasmid containing the firefly luciferase gene under the control of an ISRE-containing promotor and (2) a plasmid containing the *Renilla* luciferase gene. The cells were then treated with type I IFNs in the presence of 10% plasma or serum from patients or controls, and RLA was calculated by dividing firefly luciferase activity by *Renilla* luciferase activity. An RLA <15% of the median RLA for healthy controls was considered to correspond to neutralizing activity (dotted line; Bastard et al., 2021a). Each sample was tested once. **(D)** Correlation between ELISA and neutralization assay results for the detection of auto-Abs neutralizing type I IFNs.

We then investigated a cohort of 34 individuals infected with USUV. In 31 (20 French and 11 Italian) of these individuals, the infection was silent and detected during blood donation. Three patients (1 Hungarian [P4], 1 French [P5], and 1 Italian [P6]) had severe disease (Fig. 2 A). Samples were obtained during the first few days of infection, from the patients with severe disease. P4, P5, and P6 were all male and were 43, 78, and 80 years old, respectively. P4 presented with meningitis and was hospitalized for 7 days. P5 presented with myocarditis and systemic inflammatory response syndrome (SIRS) complicated by acute renal failure. Progression to cardiogenic shock in this patient necessitated intubation and intensive care support. P6 was hospitalized for meningoencephalitis, which progressed, resulting in death within a few days (Gaibani et al., 2023). All three cases tested positive for USUV by RT-qPCR on blood and/or urine. As described above for the patients with POWV disease, we assessed the neutralization of type I IFNs by plasma or serum from the 31 silent cases, cryopreserved whole blood from P4 and P6 (no serum or plasma samples being available for these two cases), and the serum of P5. We also included whole-blood samples from five healthy donors (without neutralizing auto-Abs) and one patient with APS-1 due to AIRE deficiency (with auto-Abs neutralizing high and low concentrations of IFN-α2, and IFN-ω, and intermediate concentrations of IFN-β) (Fig. 2, B and C) to confirm the interpretability of results obtained with whole blood. None of the silently infected individuals had detectable levels of neutralizing auto-Abs against any of the type I IFNs tested. Strikingly, samples from two of the three severe cases (P4 and P5) neutralized IFN-α2 and IFN-ω, respectively, at both high and low concentrations, and IFN-β (at an intermediate concentration for P4 and a high concentration for P5). P6 had no detectable auto-Abs and, notably, had mild COVID-19 6 mo before USUV disease. These results were consistent with the auto-Ab detection results obtained by ELISA (Fig. 2 D). Overall, none of the 31 silently infected individuals displayed detectable neutralization of type I IFNs, whereas such neutralization was observed for two of the three severe cases studied.

### Auto-Abs neutralizing IFN-α2 in the patient with the most severe RRV disease

**Figure 3.**
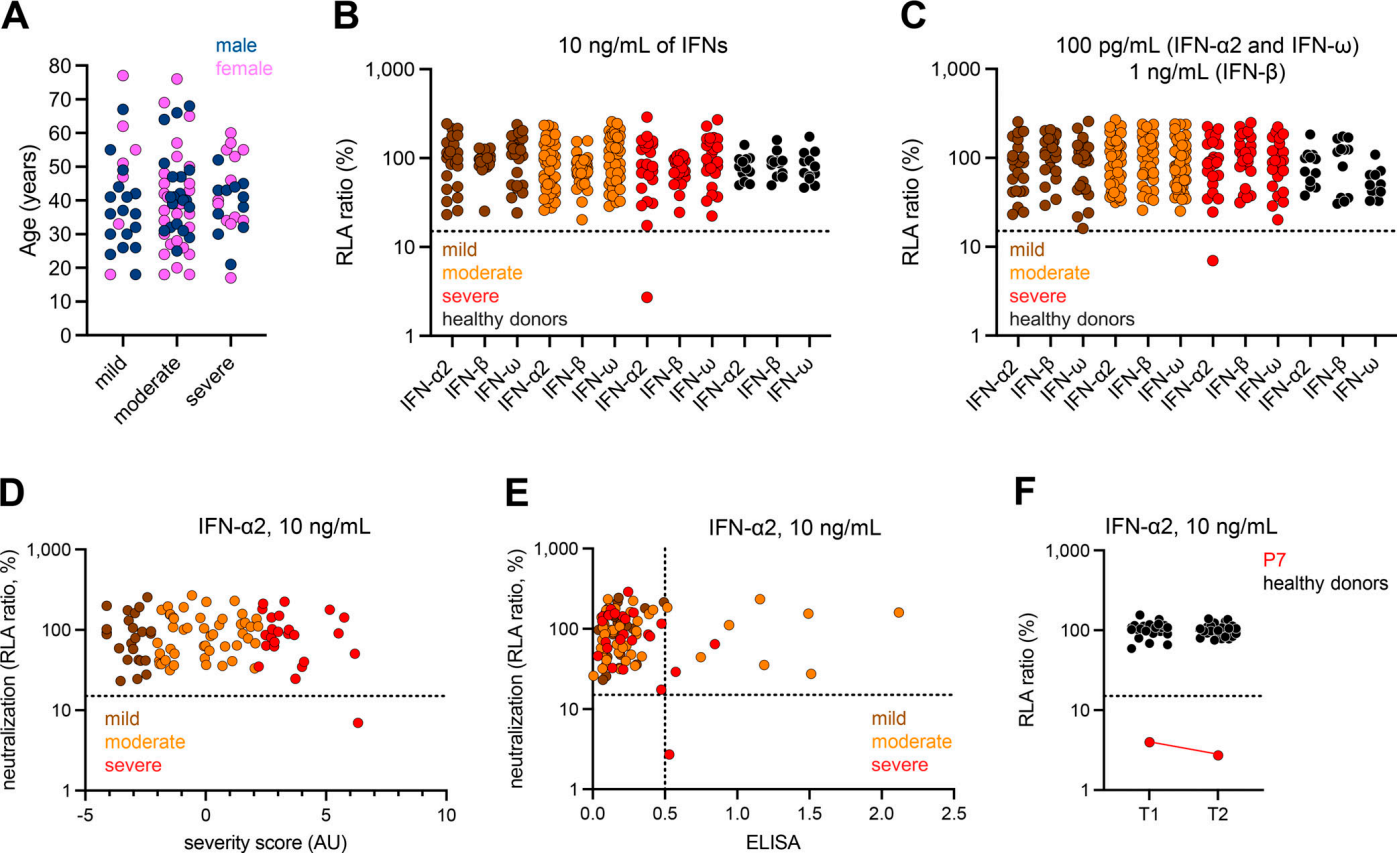
**Auto-Abs neutralizing type I IFNs in individuals infected with RRV. (A)** Age and sex distribution of the patients according to RRV disease severity. **(B and C)** Luciferase-based neutralization assay to detect auto-Abs neutralizing 10 ng/ml IFN-α2, IFN-ω, or IFN-β (B), or 100 pg/ml IFN-α2 and IFN-ω or 1 ng/ml IFN-β (C) in patients with mild (brown), moderate (orange) or severe (red) RRV disease. HEK293T cells were transfected with (1) a plasmid containing the firefly luciferase gene under the control of an ISRE-containing promotor and (2) a plasmid containing the *Renilla* luciferase gene. The cells were then treated with type I IFNs in the presence of 10% plasma or serum from patients or controls, and RLA was calculated by dividing firefly luciferase activity by *Renilla* luciferase activity. An RLA <15% of the median RLA for healthy controls was considered to correspond to neutralizing activity (dotted line; Bastard et al., 2021a). Each sample was tested once. **(D)** Correlation between IFN-α2 neutralization and the severity score of the patients. The severity score was based on the evaluation of symptoms, such as muscle pain after activity, needing to sleep longer, prolonged tiredness after activity, tired muscles after activity, headache, pains in the arms/legs, waking up tired, arms/legs feeling heavy, fevers, back pain, joint pain, and weak muscles. **(E)** Correlation between ELISA and neutralization assay results for the detection of auto-Abs neutralizing IFN-α2. **(F)** Neutralization of 10 ng/ml IFN-α2 by the original plasma sample (T1) and a longitudinal plasma sample (T2) from P7 obtained 1 year later. The HDs tested (black) were healthy individuals tested at the time of collection of each of the patient samples, but the HD samples are not longitudinal.

Finally, we investigated a cohort of 96 RRV-infected individuals from Australia. RRV infection was demonstrated by IgG seroconversion or by the detection of anti-RRV IgM and low levels of baseline-avidity anti-RRV IgG. In these patients, clinical severity was determined by calculating a severity score derived from a multidimensional reduction of the severity of prevalent symptoms (e.g., body aches, restless sleep, prolonged tiredness after activity, and febrile manifestations) by principal component analysis (Cvejic et al., 2019). Patients with a score in the top quartile were considered to have severe disease. Those with scores in the bottom quartile were considered to have mild disease, and those in between were considered to have moderately severe disease. Patients with severe disease typically missed work for a mean of 14 days (range: 2–35 days). None of the individuals with severe infection was hospitalized or died. No differences in mean age (standard deviation, SD) were observed between patients with mild (40.7 [5.1] years), moderate (40.8 [14.0] years), and severe (41.0 [10.9]) disease (Fig. 3 A). We assessed the neutralization of type I IFNs by plasma or serum from these patients with the luciferase assay described above (Fig. 3, B and C). No detectable neutralizing auto-Abs against any of the IFNs tested were detected in any of the patients with mild or moderate disease. By contrast, samples from one of the 24 patients with severe disease (P7)—a 55-year-old woman—neutralized high and low concentrations of IFN-α2. P7 had the highest severity score of the entire cohort and therefore had the most severe disease of any of the patients tested (Fig. 3 D). Interestingly, P7 was the only patient to report both headaches and fever most of the time during infection, suggesting an unusual neurotropism of RRV. P7’s auto-Abs against IFN-α2 were also detected by ELISA (Fig. 3 E), and they continued to display neutralizing activity in a follow-up sample obtained 1 year after infection, demonstrating stability over time (Fig. 3 F). Several patients with mild and moderate disease also had detectable titers of auto-Abs against IFN-α2 on ELISA, but these auto-Abs were not neutralizing (Fig. 3 E). None of the 72 individuals with mild or moderate RRV disease displayed detectable neutralization of type I IFNs. Such neutralization was observed for only one of the 24 severe cases (4.2%), the patient with the highest disease severity.

POWV and USUV infections are rare in humans and the corresponding diseases are even rarer. RRV infection is endemic to Australia and many South Pacific islands, but severe cases are also rare. Among the cases we studied, auto-Abs neutralizing type I IFNs were found to underlie the only case of severe POWV encephalitis, two of the three cases of severe USUV disease, and the most severe case of RRV disease. These auto-Abs were absent from cases of moderate POWV disease, individuals with silent USUV infection, and mild and moderate cases of RRV disease. Due to the small number of patients with each arboviral disease, we were unable to calculate the relative risk of developing severe disease conferred by auto-Abs relative to the prevalence of auto-Abs in the corresponding demographic group (Bastard et al., 2021a). However, based on previous estimates of the prevalence of these auto-Abs in the general population (Bastard et al., 2021a) and their pathogenicity in a large proportion of patients with two common arboviral diseases—WNV (40%) (Gervais et al., 2023) and TBEV (10%) encephalitis (Gervais et al., 2024b)—our current findings provide strong evidence that these auto-Abs neutralizing type I IFN may underlie severe POWV, USUV, and RRV diseases (Casanova et al., 2024; Puel et al., 2022). These auto-Abs were probably present before infection with these viruses, as in patients with life-threatening COVID-19, influenza pneumonia (Bastard et al., 2020; Zhang et al., 2022b), or WNV encephalitis (Gervais et al., 2023), and as suggested recently by an elegant longitudinal survey of a large Swiss cohort (Fernbach et al., 2024). However, it was not possible to demonstrate this unequivocally due to the absence of sample collection from the patients before infection.

These findings suggest that people at risk of producing these auto-Abs, such as patients with a history of severe viral disease (Bastard et al., 2024), autoimmunity (Beydon et al., 2022; Mathian et al., 2022), or an inborn error of self-tolerance (Bastard et al., 2021a), and elderly individuals (Bastard et al., 2021a), would benefit from testing for these auto-Abs if they inhabit or plan to travel to areas of endemicity for POWV, USUV, or RRV. Our findings also suggest that treatment with a type I IFN that is not neutralized by these auto-Abs may be beneficial in patients testing positive for these viral infections before or during hospitalization. In principle, patients with auto-Abs neutralizing IFN-α2 could potentially benefit from treatment with IFN-β, whereas those with auto-Abs neutralizing IFN-β would benefit from treatment with IFN-α2 (Bastard et al., 2021e). High doses of the antigenic IFN itself might also be considered as a potentially beneficial means of overcoming these auto-Abs, as reported for the administration of GM-CSF to patients with auto-Abs neutralizing GM-CSF in the context of pulmonary alveolar proteinosis (Tian et al., 2020). We recently developed a rapid diagnostic test that can provide results within a few hours that could be used to screen for these auto-Abs in patients admitted with suspected arboviral disease (Gervais et al., 2024a).

Auto-Abs neutralizing type I IFNs can underlie severe diseases due not only to three respiratory RNA viruses—SARS-CoV-2 (Bastard et al., 2021a, 2023, 2021c, 2020, 2022b; Puel et al., 2022), influenza virus (Zhang et al., 2022b), and MERS (Alotaibi et al., 2023)—but also six flaviviruses: YFV-17D (Bastard et al., 2022a, 2021b; Duncan et al., 2022; Hernandez et al., 2019), WNV (Gervais et al., 2023), TBEV (Gervais et al., 2024b), POWV, and USUV, and a systemic alphavirus, RRV. Unlike the other viruses mentioned, RRV is not usually neurotropic. These auto-Abs against type I IFNs may also underlie severe disease caused by other arboviruses or respiratory viruses, or even non-respiratory viruses. They might also underlie natural viral infections of organs other than the lungs and brain. However, this hypothesis seems unlikely, given the surprisingly narrow range of severe viral diseases seen in patients with autosomal recessive complete genetic deficiencies of IFNAR1 or IFNAR2 (Abolhassani et al., 2022; Bastard et al., 2022a, 2021d; Duncan et al., 2015; Duncan et al., 2022; Hernandez et al., 2019). These patients suffer mostly from adverse reactions to attenuated live viral vaccines (Hernandez et al., 2019), critical viral pneumonia (Meyts and Casanova, 2021; Abolhassani et al., 2022; Bastard et al., 2022a; Duncan et al., 2022; Zhang et al., 2022a), or encephalitis (Bastard et al., 2021d, 2022a; Meyts and Casanova, 2021). The finding of auto-Abs underlying severe WNV encephalitis, TBE, POWV, USUV, or RRV disease in turn suggests that germline genetic deficiencies of type I IFN immunity should be sought in patients with severe arboviral diseases who do not carry auto-Abs against type I IFNs.

## Materials and methods

### Patients

We enrolled an international cohort of three patients infected with POWV from the US, 40 individuals infected with USUV (20 from France and 20 from Italy), and 96 individuals infected with RRV from Australia. Written informed consent was obtained in the country of residence of each patient, in accordance with local regulations and with institutional review board (IRB) approval. Sampling was performed during acute infection for the severe POWV case and after recovery for the two patients with moderate POWV disease. The USUV patients were sampled within a week of symptom onset and the asymptomatic cases were sampled at undermined times after infection. The RRV cases were sampled a mean of 33 days (range: 6–87 days) after symptom onset, and the severe RRV cases were sampled a mean of 14 days (range: 2–35 days) after symptom onset. In P1, P2, and P3, POWV infection was identified on the basis of the detection of anti-POWV IgM in the blood (and CSF for P3) followed by a PRNT against POWV. For USUV infection, P4 was diagnosed by RT-qPCR on serum samples. P5 was diagnosed by RT-qPCR on serum samples and by the presence of neutralizing IgM anti-USUV antibodies in the serum at day 8 after symptoms onset. Samples from P6 were tested in molecular and serological assays: serum, plasma, and urine specimens were extracted from 500 µl of the sample, eluted in a volume of 55 µl and tested by multiplex real-time PCR, which revealed the presence of USUV in all sample types (Gaibani et al., 2023). The asymptomatic cases were identified on the basis of positive results for RT-qPCR on serum samples. Finally, all RRV cases were diagnosed by serological analysis revealing the presence of anti-RRV IgM antibodies. The experiments for measurement of auto-Abs to type I IFNs were conducted in France and the USA, in accordance with local regulations and guidance from the French National Agency for Medicine and Health Product Safety, the Institut National de la Santé et de la Recherche Médicale in Paris, France, and with the approval of the IRB of the Rockefeller University in New York, NY, USA, respectively.

### Luciferase reporter assay

The blocking activity of anti-IFN-α2, anti-IFN-ω, and anti-IFN-β auto-Abs was assessed in a reporter luciferase assay, as previously described (Bastard et al., 2021a). Briefly, HEK293T cells were transfected with a plasmid encoding the firefly luciferase gene under the control of the human IFN-sensitive response element (ISRE) promoter in the pGL4.45 backbone and a plasmid constitutively expressing the *Renilla* luciferase as a control for transfection (pRL-SV40). Cells were transfected in the presence of the X-tremeGene9 transfection reagent (ref. number 6365779001; Sigma-Aldrich). After 24 h, cells in Dulbecco’s modified Eagle medium (Thermo Fisher Scientific) supplemented with 2% fetal calf serum and 10% control or patient serum/plasma/whole blood (after heat inactivation at 56°C, for 20 min) were either left unstimulated or were stimulated with unglycosylated rhIFN-α2 (ref. number 130-108-984; Miltenyi Biotec), unglycosylated rhIFN-ω (ref. number 300-02J; Peprotech) at a concentration of 10 ng/ml or 100 pg/ml, or glycosylated rhIFN-β (ref. number 300-02BC; Peprotech) at a concentration of 10 or 1 ng/ml for 16 h at 37°C under an atmosphere containing 5% CO_2_. Finally, the cells were lysed by incubation with a lysis buffer (provided in ref. number E1980; Promega) for 20 min at room temperature and luciferase levels were measured with the Dual-Luciferase Reporter 1000 assay system (ref. number E1980; Promega) according to the manufacturer’s protocol. Luminescence intensity was measured with a VICTOR-X Multilabel Plate Reader (PerkinElmer Life Sciences). Firefly luciferase activity values were normalized against *Renilla* luciferase activity values. The resulting values (luciferase induction) were then normalized against the median level of induction for non-neutralizing samples and expressed as a percentage (relative luciferase activity [RLA] ratio, %). Samples were considered to be neutralizing if the RLA ratio was below 15% of the median value for controls tested on the same day.

### ELISA

ELISA was performed as previously described (Puel et al., 2008). In brief, 96-well ELISA plates (MaxiSorp; Thermo Fisher Scientific) were coated by overnight incubation at 4°C with 1 μg/ml rhIFN-α (ref. number 130-108-984; Miltenyi Biotec), rhIFN-ω (ref. number 300-02J; Peprotech), or rhIFN-β (ref. number 300-02BC; Peprotech). The plates were washed (PBS/0.005% Tween 20), blocked by incubation with the same buffer supplemented with 2% BSA, washed, and incubated with 1:50 dilutions of plasma samples from the patients or positive and negative controls for 2 h at room temperature. Each sample was tested once. Plates were thoroughly washed (PBS/0.005% Tween 20) and horseradish peroxidase–conjugated Fc-specific IgG fractions from polyclonal goat antiserum against human IgG (Nordic Immunological Laboratories) were added to a final concentration of 1 μg/ml. Plates were incubated for 1 h at room temperature and washed. The substrate was added and optical density was measured (450 nm). All the incubation steps were performed with gentle shaking (600 rpm).

## Data Availability

All data supporting the findings of this study are available within the main text and supplemental material and from the corresponding authors upon request.
